# Bergson, Pan(en)theism, and ‘Being-in-Life’

**DOI:** 10.1007/s11841-022-00933-0

**Published:** 2022-12-01

**Authors:** King-Ho Leung

**Affiliations:** St Mary’s College, University of St Andrews, South Street, St Andrews, KY16 9JU UK

**Keywords:** Henri Bergson, Metaphysics, Panentheism, Pantheism, Vitalism

## Abstract

Recent philosophy has witnessed a renewed interest in the works and ideas of Henri Bergson (1859–1941). But while contemporary scholarship has sought to rehabilitate Bergson’s insights on time, memory, consciousness, and human freedom, comparatively little attention has been paid to Bergson’s relationship to pantheism. By revisiting the ‘pantheism’ controversy surrounding Bergsonian philosophy during Bergson’s lifetime, this article argues that the panentheistic notion of ‘being-in-God’ can serve as an illuminating framework for the interpretation of Bergson’s philosophy. By examining the ‘pantheist’ readings of Bergson and comparing and contrasting Bergson’s philosophy of life with Spinoza’s panentheistic metaphysics, this paper shows that an account of ‘being-in-Life’ is key to Bergson’s metaphysical outlook as well as his account of philosophy as a practice of ‘intuitive’ thinking. In so doing, this paper highlights some of the implicit religious motifs not only in Bergson’s metaphysical outlook but also in his conception of the task of philosophy.

In the years following the publication of his most famous book, *Creative Evolution*, Henri Bergson (1859–1941) faced many fierce critiques from Christian thinkers, including from the Vatican, which accused him of pantheism (see Grogin, 1988).^1^ But while recent philosophy has witnessed a renewed and increasing interest in Bergson’s philosophy and the broader legacy of French Spiritualism, particularly Bergson’s insights on time, memory, consciousness, freedom as well as the practice of philosophy as a way of life (see Sinclair & Wolf, 2022; Sinclair, 2020; Lefebvre & Schott, 2019; Ansell-Pearson, 2018),^2^ comparatively little attention has been paid to Bergson’s relationship to pan(en)theism.^3^ By revisiting Bergson’s ‘pantheism’ controversy as well as some of the early commentaries on Bergsonian philosophy during Bergson’s lifetime, this paper seeks to offer a reading of Bergson’s relationship to pantheism (and panentheism), and in turn demonstrate how a ‘panentheistic’ reading of Bergson’s metaphysics can help to clarify the overall shape of his thought as well as his place in the history of western philosophy.

A ‘pan*en*theistic’ reading of Bergson is not new. In his survey of the history of panentheism, John W. Cooper (2013, pp. 144–147) not only speaks of Bergson as one of the great panentheist thinkers of the nineteenth century,^4^ but moreover traces his intellectual influence over subsequent panentheistic thinkers such as Pierre Teilhard de Chardin, Alfred North Whitehead, and Charles Hartshorne (Cooper, 2013, pp. 148–185). However, while there are certainly reasons why one may regard Bergson’s metaphysics as pantheist or panentheistic, it is not clear that Bergson should be regarded as a ‘pantheist’ and ‘panentheist’ in the strict sense of the term. Indeed, it is not the goal of this paper to establish Bergson as a panentheist or pantheist, nor is it its goal to ‘rescue’ Bergson from the charges of pantheism or panentheism. Instead, this paper seeks to argue that ‘panentheism’ can serve as a helpful framework or at least reference point for understanding Bergson’s philosophy, especially his early metaphysics which attracted accusations of pantheism.^5^ Section one of this paper provides a reading of the ‘pantheism’ controversy surrounding Bergson’s philosophy. This is followed by section two which offers an alternative reading of Bergson’s metaphysics of life in relation to Spinoza’s ontology of ‘being-in-God’. After this, section three demonstrates how the panentheistic conception of ‘being-in-God’ can provide an insightful lens for interpreting Bergson’s metaphysics as an account of ‘being-in-Life’ and its implication for the practice of philosophy.

## ‘God’

In 1914, *Creative Evolution* (1907) was placed on the Roman Catholic Church’s *Index* of prohibited books, alongside Bergson’s earlier works, *Time and Free Will* (1889) and *Matter and Memory* (1896).^6^ In the years following the publication of *Creative Evolution* and leading up to the Catholic prohibition of Bergson’s three major books, a number of notable Catholic philosophers and theologians from a wide theological spectrum—from the ‘existential Thomist’ philosopher Jacques Maritain to the neo-Scholastic Thomist manualist theologian Réginald Garrigou-Lagrange—presented fierce critiques of Bergson’s metaphysics in *Creative Evolution* as a version of ‘pantheism’.^7^

But despite the charges of pantheism and heresy, as a number of commentators have observed (see Kołakowski, 1985, p. 61; de Warren, 2010, p. 184), Bergson articulates his own view of ‘God’ only in one sentence in *Creative Evolution*.^8^ The sentence appears in a long paragraph where Bergson presents his view that reality consists of actions, changes, and movements:

It is natural to our intellect, whose function is essentially practical, made to present to us things and states rather than changes and acts. But things and states are only views, taken by our mind, of [the reality of] becoming. There are no things, there are only actions… [But there is] a centre from which worlds shoot out like rockets in a fire-works display—provided, however, that I do not present this centre as a *thing*, but as a continuity of shooting out. God thus defined, has nothing of the already made; He is unceasing life, action, freedom [*Dieu, ainsi défini, n’a rîen de tout fait; il est vie incessante, action, liberté*]. Creation, so conceived, is not a mystery; we experience it in ourselves when we act freely… In reality, life is a movement, materiality is the inverse movement, and each of these two movements is simple, the matter which forms a world being an undivided flux, and undivided also the life that runs through it. (Bergson, 1928, pp. 261–263)

In this important passage, and throughout *Creative Evolution* more broadly, Bergson argues that while it is ‘natural’ for our rational intellect to perceive the world in terms of ‘things’ and ‘states’ (or even stasis), reality in itself consists only of actions and changes: ‘things’ are only the appearance perceived by our intellect, the reality behind the appearance of things and states is not some static substance or principle of ‘being’, but a flux of ‘becoming’.^9^ As Bergson (1946, p. 119) puts it: ‘Movement is reality itself.’

While this reality of becoming consists of actions and changes, Bergson postulates that there are two inverse tendencies or movements at work in this reality: namely, life and matter. Life and matter are not two ‘things’ (not least because Bergson insists that ‘there are no things’ in reality), but ‘two movements or two tendencies across a single infinitely divisible spectrum’: Matter is a tendency of life, just as life is a tendency of matter (see Sinclair, 2020, p. 222). Put simply, matter is what we find when the movement of life relaxes and slackens, but when matter accelerates and intensifies it becomes life.^10^

Before we turn to a further analysis of Bergson’s metaphysics of ‘life’ below, let us assess some reasons why his characterisation of God would attract charges of ‘pantheism’ in light of his account of life and movement summarised above. First, while Bergson’s proposal that God is no *thing* may address the ‘onto-theological’ worries of treating God not as Being itself but as a finite ‘thing’ or a being among beings—thereby upholding an ontological difference between God and finite created beings, his portrayal of both God and reality in terms of ‘action’ raises some questions about the ontological distinction between God and creaturely being. For if all reality is a flux of becoming which consists only of ‘actions’, and that God in Godself is ‘action’ and ‘life’, and that ‘life’ is itself a ‘movement’, it sounds as if Bergson’s God is nothing more than *part of* the reality of becoming or is at best identical to the very reality of becoming itself. Second, if God *is* becoming or *part of* becoming, that means God is not the immutable eternal being (or even ‘Being’) as conceived in traditional Catholic doctrine, which frames the ontological distinction between God and creation in the terms of the broadly Platonic distinction between (eternal) being and (temporal) becoming.^11^

Following Bergson’s association of the reality of becoming with the flow of durational time (Bergson, 1991, pp. 138–39, 149–51; cf. Bergson, 1913, pp. 130, 231), Kołakowski presents this reading of Bergson’s God as ‘becoming itself’ or part of becoming in terms of time:

[Bergson’s] God is time-bound, or rather, he is time itself, and our time mysteriously participates in his while not being a mere aspect of it… Consequently, God cannot be an absolute in the sense which the Christian God is. The absolute God is timeless, he lives in the eternal present… to deny this is to destroy his wholeness, his unity, and his perfect self-containment. (Kołakowski, 1985, p. 62)

In addition to this, Kołakowski (1985, p. 63) notes that characterising God as the ‘centre’ of creativity means that Bergson’s God ‘cannot, by even the greatest effort of abstraction, be conceived apart from the world’ for there is ‘no way that the producer could be conceptually grasped as being alone, without relation to his products’.^12^ As such, Kołakowski (1985, p. 63) suggests that ‘the label “pantheism” is not inappropriate’ for Bergson’s philosophical vision in *Creation Evolution*.

While there are ways in which Bergson’s philosophical vision could be interpreted as a kind of pantheism, as a number of commentators have pointed out (see Chevalier, 1928, pp. 254–55n41, 269–71, 274–75; Gunn, 1920, pp. 128–9; Kołakowski, 1985, p. 65; de Warren, 2010, p. 185; Grogin, 1988, p. 143), in his letters to his Catholic critic Fr Joseph de Tonquédec, Bergson makes it clear that the principle of life or *élan vital* (vital impulse) depicted in *Creative Evolution* is emphatically not God.^13^ As Bergson writes in his letter dated to 20 February 1912:

I speak of God as the source whence issue successively, by an effort of his freedom, the currents or impulses each of which will make a world; he therefore remains distinct from them… Now the considerations set forth in my *Essai sur les données immédiates* [*Time and Free Will*] result in bringing to light the fact of freedom, those of *Matière et Mémoire* [*Matter and Memory*] point directly, to the reality of Spirit, those of *L’Évolution créatrice* [*Creative Evolution*] exhibit creation as a fact. From all this emerges clearly the idea of a God, creator and free, the generator of both Matter and Life, whose work of creation is continued on the side of Life by the evolution of species and the building up of human personalities. From all this emerges a refutation of monism and of pantheism. (Bergson, as quoted and translated in Gunn, 1920, pp. 128–29; cf. Bergson, 1972, pp. 963–64)

However, as J. Alexander Gunn points out in his early commentary on Bergson’s philosophy, even if Bergson’s outlook may not strictly be a pantheism, his account of God still differs from the traditional Catholic conception:

[F]or Catholic theology, God is not merely the source from which the river springs, God does not develop Himself to a world but He causes it to appear by a kind of creation quite different from that of Bergson… For Bergson, God is a Being immanent in the universe… He is absolutely unfinished, not complete or perfect… He is not to be conceived as existing apart from and independent of the world…[Nonetheless] Bergson’s God is not the God of pantheism, because, for him, the Deity is immanent in nature, [but] not identifiable with it. (Gunn, 1920, p. 129)

While Bergson’s philosophy may not be a pantheism insofar as his God is ‘not identifiable’ with the world, to the extent that this God is still deeply ‘immanent in’ the world and ‘cannot’, as Kołakowski (1985, p. 62) says, ‘be conceived apart from the world’, Bergson’s outlook may be regarded more properly as a ‘pan*en*theism’. Further to explore this point, let us examine the distinction between pantheism and pan*en*theism by looking at Spinoza, whose metaphysics is often named in the debates over whether Bergson’s philosophy is pantheistic.^14^

## Pan(en)theism

Although Spinoza is commonly accused of being a ‘pantheist’ with his metaphysical monist identification of God with ‘Nature’, in her recent work on *Spinoza’s Religion* Clare Carlisle (2021, esp. pp. 56–78) argues that the seventeenth-century philosopher ought to be regarded more properly as a ‘pan*en*theist’.^15^ As Carlisle (2021, p. 63) notes, whereas pantheism sees God as identical with the universe—that ‘the doctrine that God is everything and everything is God’, panentheism is by contrast the view that ‘whatever is, is *in* God’: ‘While pantheism denies God’s difference from the world, panentheism affirms this difference.’ Accordingly, rather than a pantheist who sees ‘being *as* God’ or that ‘being *is* God’, it is more appropriate to see Spinoza as a ‘pan*en*theist’ who articulates an ontological vision of ‘being-*in-*God’,^16^ for his metaphysics ‘establishes an asymmetry between God and the universe, which is confirmed by his distinctive use of the concepts of substance and mode’ (Carlisle, 2021, p. 67).

Indeed, at the outset of part I of the *Ethics*, Spinoza begins with the axiom that ‘Whatever is, is either in itself or in another’ (*Ethics*, 1a1). Following the Aristotelian conception of substance made popular by medieval scholastic theologians such as Thomas Aquinas, Spinoza defines substance as ‘that which is in itself’ (*in se est*) and a mode as ‘that which is in another’ (*in alio est*) (*Ethics*, 1d3, 1d5). Echoing the scholastic teaching of divine aseity that God only is self-subsistent being—that God alone exists ‘in itself’ (*in se*) (cf. Aquinas, *Summa Theologiae*, I.3–4), Spinoza posits that God is the one and only substance in existence (*Ethics*, 1p14), whereas all other things are modes which exist ‘*in*’ this substance *qua* God, with the ontological status of ‘being-in-God’ (Carlisle, 2021, p. 57).

As Carlisle points out, Spinoza’s ontology of ‘being-in-God’ is broadly in continuation with the conceptual structure of Christian theological metaphysics of participation, especially as expounded by Aquinas. For just as Aquinas posits that ‘God is essential being’ while ‘all beings apart from God are not their own being, but are beings by participation [*per participationem*]’ (*Summa Theologiae* I.1.3, *ad* 3), Spinoza draws a similar ontological distinction between God who is substance ‘in itself’ and all other things which are modes of substance which exist only ‘in another’. To this extent, Carlisle (2021, p. 101) argues that ‘Aquinas’s concept of participation does similar metaphysical work to Spinoza’s concepts of substance and mode, in distinguishing between what exists by virtue of its own being (i.e., God) and what exists “by participation” (i.e., created things).’

Like Aquinas who follows Augustine in affirming that ‘all things are in God inasmuch as they are contained by God’ (*Summa Theologiae*, I.8.1, *ad* 2), Spinoza teaches that all created things *qua* finite modes only exist by way of a certain sharing or ‘participation’ *in* the one self-subsistent being or substance that he calls ‘God’ (see Carlisle, 2021, pp. 101–104).^17^ As a being can only ‘participate’ in something that it is *not*—as Spinoza says, ‘*in* another’, there is an irreducible ontological difference between God *qua* self-subsisting substance and finite creatures *qua* modes: Spinoza’s ontology is accordingly not a pantheism but a pan*en*theism insofar as it upholds this ontological difference.

In light of this, one may note a ‘participatory’ parallel between this account of being-*in*-God and Kołakowski’s characterisation of Bergson’s God as time itself: not unlike how created things exist by ‘a certain participation’ (*quaedam participatio*) in God’s being or in God who *is* being itself in the Thomistic schema (see Aquinas, *Compendium Theologiae*, part 1, §135), in Kołakowski’s (1985, p. 62) reading of Bergson our time ‘mysteriously participates in’ God’s time or indeed in God who *is* time itself. One obvious issue with drawing such a parallel would be that in Christianity, time itself is not uncreated but a part of creation. So, like other creatures, time has being only by participation in God’s uncreated and eternal being which, according to Aquinas, ‘surpasses all time’ (*Summa Theologiae*, II-II.12.2, *ad* 2). However, Aquinas’s account of ‘analogy’ in his articulation of the ontological relationship between God and creation could also provide ways to make sense of the Bergsonian notion of the creaturely participation in God who is time.^18^

In Aquinas’s discussion of properties that are possessed by both God and creatures, in cases where the properties are ‘perfections’ that are essentially possessed by God, such as goodness, the ‘goodness’ possessed by creatures can be understood only in *analogical* sense: the goodness of creatures is only a distant likeness to God’s perfect goodness (see Aquinas, *Summa Contra Gentiles*, I.28–34, cf. I.37–38). For whereas creatures *have* goodness, God *is* goodness or indeed *the* Good itself: creatures have goodness only by ‘a certain diminished participation’ in God’s essential goodness, because ‘belongs to God absolutely, but not to the creature’ (Aquinas, *Summa Contra Gentiles*, I.29.5). Similarly, insofar as God is said to be ‘Life itself’ (Aquinas, *Summa Contra Gentiles*, I.97–98; *Summa Theologiae*, I.18), and that ‘to live belong to God in a supreme way’ (Aquinas, *Summa Contra Gentiles*, I.97.3), creatures have life only by participating *in* God’s Life (see Aquinas, *Summa Theologiae*, I.18.4, esp. *ad* 4; cf. *Summa Contra Gentiles*, I.98.4).^19^ Given Bergson’s statement that God is ‘life’ and ‘action’ in *Creative Evolution*, and that God is ‘the source’ of ‘the currents or impulses [which] make a world’ and ‘therefore remains distinct from them’ in his 1912 letter, we may note that the way in which Bergson’s God is ‘life’ and ‘action’ is indeed *distinct from* the way in which the world is constituted by the movement and action of ‘life’. Accordingly, to the extent God and the world are both said to be ‘life’ but are nonetheless distinct from each other - as how God and creation are both said to be ‘good’ but in different ways according to Aquinas’s account of analogy, one may say that there is an ‘analogy of life’ at work in Bergson’s metaphysics. By extension, in light of the close affinity Bergson sees between ‘life’ and ‘time’, one can moreover say that finite beings—or, more accurately, finite movements or actions—exist only by *having* time in an analogical manner to the way in which God exists as the one who *is* time itself: indeed, one may even say that finite reality only exist as a temporal flow of becoming by participating in God who *is* ‘time’, ‘action’ and ‘incessant life’ in itself.

## Being-in-Life

But what does it mean to participate in such a ‘God’ of becoming who is nothing other than ‘action’ and ‘unceasing life’? What does this speculative metaphysical outlook entail for the way we live *life*? In Spinoza’s panentheistic schema of ‘being-in-God’, participation in the divine is not just an ontological thesis but, as Carlisle (2021, p. 92) notes, an ‘empowering, joyful religious attitude’. As we shall see with reference to Bergson, there is also a sense of religiosity underlying his ontology of movement and becoming.

As noted above, Bergson (1928, p. 261) argues that we ‘naturally’ tend to perceive reality in static terms of things and objects—or indeed in terms of ‘matter’ instead of ‘life’—for ‘essentially practical’ reasons: that such static ‘intellectual’ perception enables us to manipulate and profit from things and objects in our everyday life (see Sinclair, 2020, p. 166). For Bergson, the conception of God as ‘incessant life’ and ‘action’ involves a change in how we comprehend the world: a conversion from our ‘natural’ perception in terms of static things and objects to a new understanding of reality in terms of dynamic movements and actions—or indeed, in terms of *life*. As Bergson notes:

What is required is that we should break with certain habits of thinking and perceiving that have become natural to us. We must return to the direct perception of change and mobility. (Bergson, 1946, p. 118)

As opposed to perceiving and thinking about the world in terms of static things and objects instead of change and movement—or indeed in terms of ‘matter’ instead of ‘life,’ Bergson argues that we should ‘*reverse* the normal direction of the workings of thought’ and attune ourselves to ‘the very movement of the inner life of things’ (Bergson, 1946, p. 160, emphasis added).

While Bergson’s arguments for this ‘reversal’ of our normal direction of thought or the ‘return’ to the direct perception of change and movement are presented strictly in philosophical terms, several of his early commentators have compared Bergson’s conceptual schema to religious and theological accounts of conversion and salvation.^20^ For instance, although Bergson speaks of ‘intellectual’ static perception as a ‘habit of thinking and perceiving’ that we can break, Harald Høffding argues in his 1913 lectures on Bergson that the Bergsonian conception of the ‘intellect’ or ‘intelligence’ is comparable to the Christian notion of ‘original sin’:

It seems to Bergson that there has been a sort of original sin. Misled by the type of jargon that is produced by the practical life, we have turned our backs upon the immediate given, and have devoted ourselves to the abstractions and divisions of reflection… The original sin was committed when intelligence replaced instinct. Instinct is nearer to life than intelligence. (Høffding, 1915, pp. 248–249)

The type of ‘instinct’ that Bergson advocates in the ‘reversal’ and ‘return’ he envisions—not dissimilar to traditional religious concepts of ‘conversion’ or ‘repentance’—is not simply the type of basic vital or animal instinct that humans share with other living beings (see Bergson, 1928, p. 151). It is rather, as Høffding (1915, p. 250) points out, a ‘disinterested instinct’ where one’s vital instinct ‘become[s] disinterested and free[s] itself from its servitude to a practical end’.

This is what Bergson calls ‘intuition’. As consciously ‘disinterested’ or even self-reflective instinct, intuition is something that conscious human beings need to cultivate as rational and intellectual living beings. As J. Alexander Gunn writes in his 1920 commentary on Bergson:

We should be led into the very interior of Life by Intuition, that is, by Instinct become disinterested, conscious of itself, capable of reflecting on its object and enlarging it indefinitely. In proclaiming the gospel of Intuition, Bergson’s main point is to show that man is capable of an experience and a knowledge deeper than that which the Intellect can possibly give. (Gunn, 1920, p. 102)

In this regard, what Gunn (1920, pp. 98–109) calls Bergson’s ‘gospel of intuition’ may be seen as the quasi-religious answer to, in Høffding’s (1915, p. 251) description of Bergson, ‘the question whether there is not some hope of redemption after the original sin’ of our ‘natural’ intellectual habits of perceiving reality.^21^ Indeed, in Gunn’s reading of Bergson’s ‘gospel’, the possession of intuition is comparable to the Christian soteriological concepts of union with God or even ‘atonement’:

[B]y Intuition we shall find ourselves in—to invent a word—‘intunation’ with the *élan vital*, with the Evolution of the whole universe, and this absolute feeling of ‘at-one-ment’ with the universe will result in that emotional synthesis which is deep Joy. (Gunn, 1920, p. 109)

While Gunn refers here to union with the *élan vital* instead of God, to the extent that this union is also a quasi-religious ‘“at-one-ment” with the universe’ itself and that the *élan vital* is often seen to be simply identical to Bergson’s conception of God, here we can detect a pan*en*theistic structure in Bergson’s ‘intuitive’ account of one’s attainment of a joyful or even beatific union with the universe or indeed the *élan vital*—not unlike the ‘boundless joy’ of being in ‘union with God’ affirmatively depicted by Bergson some years later in *The Two Sources of Morality and Religion* (1932).^22^

This quasi-religious or even quasi-panentheistic reading of the ‘intuitive’ goal to unite oneself with the universe or with ‘nature’ can be confirmed by Bergson’s very own account of the task of philosophy in his lecture on ‘Philosophical Intuition’, given at the Philosophical Congress in Bologna in April 1911: ‘[It] belongs to philosophy… to follow the moving reality, adopt the becoming which is the life of things… The philosopher… seeks to be at one with nature’ (Bergson, 1946, p. 104). As Bergson notes in this lecture, ‘to be one with nature’ and ‘adopt the becoming which is *the life of things*’ is to find *life* in all things—even inanimate ‘dead’ things:

[L]et us grasp afresh the external world as it really is… let us in a word become accustomed to see all things *sub specie durationis*: immediately in our galvanized perception what is taut becomes relaxed, what is dormant awakens, what is dead comes to life again… [P]hilosophy thus understood will offer to all of us, at all times, by breathing life once again into the phantoms which surround us and by revivifying us. (Bergson, 1946, p. 106)

As though echoing the Jesuit ethos of finding God in all things, Bergson’s ‘intuitive’ vision is a quasi-spiritual outlook which seeks to find life in all things.

This somewhat panvitalistic outlook is notably articulated in the powerful conclusion of Bergson’s lectures on ‘The Perception of Change’ delivered at Oxford in May 1911 (shortly after Bologna). To quote it at some length:

Everything comes to life around us, everything is revivified in us. A great impulse carries beings and things along. We feel ourselves uplifted, carried away, borne along by it. We are more fully alive [with] this increase of life… In fact, the more we accustom ourselves to think and to perceive all things *sub specie durationis*, the more we plunge into real duration. And the more we immerse ourselves in it, the more we set ourselves back in the direction of the principle, though it be transcendent, in which we participate [*participons*] and whose eternity is not to be an eternity of immutability, but an eternity of life: how, otherwise, could we live and move in it? *In ea vivimus et movemur et sumus*. (Bergson, 1946, pp. 131–132)

What we find in this striking passage is a participatory ontology of ‘being*-in*’ with some interesting parallels to the pan*en*theistic notion of ‘being-in-God’. For Bergson, the goal of philosophical ‘intuition’—or even of philosophy per se—is to plunge or immerse oneself ‘*in*’ the flow of becoming which he calls real duration.^23^ Indeed, Bergson even characterises ‘intuition’ in terms of a *participation in* some ‘transcendent principle’ which very much resembles the traditional religious notion of ‘God’.

But as opposed to the traditional theological conception of God, Bergson’s account of the transcendent principle in which we participate is explicitly *not* immutable but is instead a principle of infinite mutability which Bergson names ‘life’.^24^ Paraphrasing St Paul’s saying that ‘in God we live and move and have our being’ (Acts 17:28; translated into Latin in the Vulgate as ‘*In ipso enim vivimus, et movemur, et sumus*’), Bergson submits that it is this ever mutable ‘eternity of life’ in which ‘we live and move and have our being’: *In ea vivimus et movemur et sumus*. As such, what we find in Bergson’s outlook is proper speaking not so much a panvitalism or a pan(en)*the*ism, but rather what may be more accurately called a ‘pan-*en*-vitalist’ ontology of ‘being-*in*-Life’. Because for Bergson, it is by virtue of our participation in ‘Life’—and not ‘God’, at least as traditionally conceived—that we possess life, movement, and being.^25^

However, it is worth noting that while Bergson argues that we only have life, movement and even being by participation in the transcendent principle of ‘Life’, his key point in the passage quoted above is not that one participates in ‘Life’ by ‘plunging’ or ‘immersing’ oneself in the becoming of real duration through intuition. To plunge or immerse oneself ‘intuitively’ in real duration is not simply identical to one’s ‘participation’ in Life, it is rather to ‘set’ oneself ‘back in the direction of the principle [of Life]’. In other words, ‘intuition’ itself is not ‘participation’, but the orientation of one’s attention or even one’s being to the ‘vision of universal becoming’ and ‘make it penetrate into our everyday life’ (Bergson, 1946, p. 141). Indeed, for Bergson (1946, p. 108), to perceive reality through the lens of universal becoming is to not only find *life* in all things, but moreover to bring about a ‘transfiguration’ of our everyday life: ‘Not only would philosophy gain by it, but our everyday life… would perhaps be transformed and, as it were, transfigured [*transfigurées*]’.

## Concluding Remarks

Commenting on the allegations of ‘pantheism’ against Bergson, Nicolas de Warren (2010, p. 184) notes that ‘Bergson’s presumed pantheism hinges on accepting [an] interpretation of God’s immanence in the manifold of creative evolution’ which presupposes a ‘*strong* identification of God with the vital impulse [*élan vital*]’ (emphasis in original). However, as Bergson makes clear in his aforementioned letters to de Tonquédec, his account of God and creation in *Creative Evolution* is by no means a pantheist thesis, but rather ‘a refutation of monism and of pantheism’.^26^ Moreover, as Bergson argues in his correspondence with de Tonquédec, his ‘refutation’ of monism and pantheism in *Creative Evolution* is specifically ‘aimed at the Spinozist conception of being’.^27^ However, despite Bergson explicit attempts to distance himself from Spinoza and pantheism, his Catholics critics such as Jacques Maritain remain convinced.^28^ Indeed, while Maritain admits that ‘Bergson is not a pantheist in the way that Spinoza was’, he argues that although ‘Bergson does not intend to be pantheistic’, by positing that ‘[all things] are one, not in Spinozist *substance*, but in *pure becoming*’, ‘Bergsonian metaphysics in spite of itself, falls a prey to pantheism’ (Maritain, 1955, pp. 199–200, 320).

It is not the goal of this paper to advocate for a ‘*strong* identification of God with the vital impulse’ which de Warren finds in critical readings of Bergsonian philosophy as a pantheism. Instead, by re-reading Bergson’s metaphysical outlook in light of Clare Carlisle’s recent reading of Spinoza not as a pantheist but a pan*en*theist who espouses an ontology of ‘being-in-God’, this paper has sought to highlight how Bergson’s philosophy of ‘being-in-Life’ resembles a panentheistic ontology which sees all things as being ‘*in*’—or even *participating in*—a transcendent divine principle. But as opposed to a ‘static’ account of an eternally immutable substance named ‘God’ (as traditionally associated with Spinozist metaphysics), Bergson’s conception of the transcendent principle in which all things participate is an eternally mutable—and indeed mutating—movement of *life*.^29^ While Bergson notably names this transcendent principle ‘Life’ instead of ‘God’ and emphatically says that God in his conception ‘remains distinct from’ the vital currents and movements that constitute the (created) world,^30^ the way in which he presents this transcendent principle of Life ‘in which we live and move and have our being’ explicitly echoes the traditional Christian biblical account of God as the one in whom ‘we live and move and have our being’ (Bergson, 1946, p. 132). To this extent, one can indeed see Bergson’s philosophical metaphysics as a quasi-theological vitalism which broadly follows the traditional religious conception of God as Life itself.^31^

As we saw with reference to a number of early commentaries, Bergson’s account of the task and practice of philosophy has many elements which resemble the notions of ‘original sin’, ‘conversion’, and ‘salvation’ one normally associates with religious life. With this quasi-religious conception of philosophy as a spiritual exercise of aligning one’s mind and being to the transcendent principle of ‘Life’, which Bergson envisions as a practice that ‘vivifies’ oneself or even ‘transfigures’ one’s everyday life, Bergson’s philosophy of life is at once a speculative metaphysics of life *and* an engaged way of living—not unlike the twofold ontological *and* ethical account of ‘being-in-God’ in Spinoza’s *Ethics*.^32^ Reading Bergson’s philosophy of ‘being-in-Life’ in light of Spinoza’s ‘being-in-God’ not only illuminates Bergson’s relationship to historical construals of pantheism and panentheism,^33^ as well as the correlation between the metaphysical-ontological and the ethical-existential aspects of Bergsonian philosophy,^34^ it can moreover show us how the panentheistic notion of ‘being-in-God’ can provide a conceptual framework to analyse ideas and outlooks of philosophers and thinkers who are not strictly panentheist or immediately associated with panentheism or pantheism.

## Footnotes

1 For instance, Charles Corbière argued in 1910 that ‘Bergson’s conception [of God] leads to pantheism’ (Corbière, 1910, as quoted and translated in de Warren, 2010, p. 184).

2 Note also the recent English translations of studies on Bergsonian philosophy such as Jankélévitch, 2015; Péguy, 2019.

3 For instance, the notions of ‘pantheism’ and ‘panentheism’ are not mentioned at all in Jennifer Newsome Martin’s (2021) recent article on Bergson’s influence on the *Ressourcement* movement in twentieth-century Catholic theology as well as Catholic critiques of Bergson.

4 See also Cooper, 2013, p. 120: ‘[Some of] the most interesting and influential figures in Germany, England, the United States, and France who adapted and modified the panentheistic theologies of Schelling and Hegel… such as Coleridge, Emerson, Peirce, James, and Bergson, are famous figures in literature or philosophy, although their panentheism may not be common knowledge.’

5 While this paper focuses primarily on Bergson’s earlier work on metaphysics as well as some of the early commentaries on his philosophy from the first two decades of the twentieth century, some allusions will also be made to his later 1932 book on religion, ethics, and politics, *The Two Sources of Morality and Religion* (see footnotes 22 and 24).

6 For a selection and discussion of quotes from the reports by the Catholic Church’s Congregation for the Doctrine of the Faith which condemned these works, see Neveu, 2003.

7 See Maritain, 1955; Garrigou-Lagrange, 2021, esp. pp. 156–160, 189–202. Maritain’s critique of Bergson is further discussed in the conclusion of this paper. While Maritain and Garrigou-Lagrange were both committed Catholics, they famously differed in their political orientations. In light of Bergson’s Polish-Jewish ancestry—and the connections made to Spinoza (also notably Jewish) in the accusations of pantheism, it may be noted that Garrigou-Lagrange—the leading conservative Vichy-supporting neo-scholastic theologian in Rome—is often regarded as an anti-Semite. See Cummings, 2021, p. 172; cf. Peddicord, 2005, pp. 80–113.

8 In Bergson’s two other books that are banned by the Catholic Church, the word ‘God’ appears only once (in a passing discussion of Leibniz’s determinism) in *Time and Free Will* and not even once in *Matter and Memory*. See Bergson, 1913, p. 214.

9 As opposed to ‘intelligence’ or the intellect which perceives in terms only of things and stasis, Bergson suggests that ‘intuition’ is what attunes the human being to the reality of change and becoming. On Bergson’s distinction between ‘intuition’ and ‘intelligence’, see Sinclair, 2020, pp. 157–174.

10 As Sinclair (2020, pp. 127, 219–220) points out, Bergson is not entirely consistent on this point: whereas in *Creative Evolution* extended matter is said to be the relaxation or ‘*ex-*tension’ of life and life the acceleration or *in*tensification of matter, earlier in *Matter and Memory* matter is said to be the intensification or contraction of consciousness (which Bergson associates with life in *Creative Evolution*, while consciousness is the dilation or relaxation of matter.)

11 This is precisely the argument made by the neo-scholastic Fr Édouard Hugon in his 8 March 1913 letter to the Catholic Church’s Secretary of the Congregation of Faith to recommend the inclusion of Bergson’s works on the Index prohibited books. See Neveu, 2003, p. 544.

12 Kołakowski (1985, p. 63) further adds: ‘To be sure, Aquinas’s God is also known to us by the intermediary of his creatures… none the less [for Aquinas] we know a priori that there is no ontologically necessary link between God and the world. And yet to Bergson this link is ontological.’ The relationship between Aquinas’s and Bergson’s metaphysics is further discussed below.

13 This is also acknowledged by Maritain (1955, pp. 92n1, 186–187, 199), although he remains unconvinced by Bergson’s claims in his letters to de Tonquédec. In addition to the distinction between the *élan vital* and God, Bergson also distinguishes between different types of *élan* in *Creative Evolution* (e.g., *l’élan originel*, *l’élan commun*, and *l’élan primitif du tout*), however, we shall focus on the *élan vital* in the remainder of this essay. I would like to thank one of the anonymous reviewers for this point.

14 However, it is worth noting that in these allusions Bergson’s position is often contrasted with Spinoza’s, for example, see Kołakowski, 1985, p. 62: ‘Bergson’s God may seem more comprehensible to us than the God of Christian theologians because we can imagine him as a real person, and we are unable to conceive of a personality without time. The description of an absolute, self-contained, and timeless being strongly suggests Spinoza’s God, who cannot share the characteristics of a person. In this sense it may be argued that the label “pantheism”, which Christian critics have so often tagged on to Bergson’s philosophy, is unjustified.’

15 As Carlisle (2021, pp. 63–64) points out, the reading of Spinoza as a pantheist often rests on his celebrated phrase ‘God or Nature’ (*Deus sive Natura*) in the *Ethics* (Part IV, Preface), but such readings sometimes overlook the fact that ‘Spinoza distinguishes between *natura naturans* and *natura naturata*, and [only] equates *natura naturans* with God… *natura naturans* and *natura naturata* are not two distinct objects; nevertheless, they are not simply alternative labels for the same reality. The distinction between them signifies an ontological difference… *natura naturata* is “in” *natura naturans*, and dependent on it’ (referencing Spinoza, *Ethics*, 1p29s and 1p15).

16 Drawing on Spinoza’s dictum that ‘*Whatever is, is in God*’ (*Ethics*, 1p15), Carlisle (2021, see esp. pp. 56–78, 92–107) argues that ‘being-in-God’ is the key ontological motif for understanding Spinoza’s metaphysics (and religious outlook). Cf. Carlisle, 2021, p. 57: ‘Étienne Souriau [2015, pp. 190–91] observed that “the meaning of the little word *in* as it is found in [the first axiom of the *Ethics*] is the key to all of Spinozism”, and more recently Don Garrett [2008, p. 10] suggested that “being *in*” is “the most fundamental relation in Spinoza’s metaphysics”.’

17 As Carlisle (2021, p. 101) recognises, to highlight the similarities between Aquinas and Spinoza ‘is certainly not to deny that Aquinas’s metaphysics—and not least his doctrine of creation—is fundamentally different from Spinoza’s in several important respects’. For instance, unlike Spinoza, Aquinas insists that God cannot be said to be ‘substance’ for that would place God in a genus or category (as Aquinas wants to attribute ‘substantiality’ to beings other than God). See Aquinas, *Summa Theologiae*, I.3.5, *ad* 1. As Carlisle points out, Spinoza’s own use of the word ‘participating’ (*participat*) refers not to participation in the being (*esse*) or substance of God, but to God’s divine nature or *natura* (Spinoza, *Ethics*, 4p45c2s), as expression of participating in divine joy or indeed blessedness (Carlisle, 2021, pp. 92–100). In this regard, Spinoza’s account of participation in the divine nature not only clearly echoes 2 Peter 1:4 (‘to become participants of the divine nature’), but also Aquinas’s teaching that the Christian beatific life of grace as ‘participation in the divine nature’ (*participatio divinae naturae*). See Aquinas, *Summa Theologiae*, I-II.112.1. One might further note here that the way in which Spinoza’s ‘participation’ refers to the divine nature (*natura*) as opposed to the divine being or essence (*esse*) is in line with traditional Christian formulations which serve to safeguard the ontological difference between God and creature (see Crisp, 2019, pp. 201–202).

18 Cf. Carlisle’s (2021, p. 222n5) provocative claim that whereas ‘the *univocity of being* is proper to pantheism… the *analogy of being* is proper to panentheism, as expressing both difference and relation: the relation of being-in-God’ in her critique of Deleuze’s (1990) influential reading of Spinoza as a pantheist and a chief advocate of the univocity of being, according to which all beings, including God, possess ‘being’ in one and the same way.

19 To the extent that God’s being *is* Life itself, and that everything has their existence by participation in God’s being, then we might say that all beings—living and non-living alike—participate in God’s Life, see Leget, 1997, pp. 46–47: ‘Since God’s essence is life, all creatures are life in God… living and non-living creatures share in the life of God according to an incomprehensible mode of being which is God’s “mode of being”… All created beings participate in His life.’

20 Note that the following discussion does not rely on explicitly religious interpretations (or even appropriations) of Bergson’s philosophy such as Chevalier, 1928.

21 The ‘gospel of intuition’ is the title of Gunn’s (1920, pp. 98–109) chapter on Bergsonian intuition in his commentary.

22 As Bergson (1977, p. 219) writes in *The Two Sources*: ‘however close the union with God may be, it could be final only if it were total. Gone, doubtless, is the distance between the thought and the object of the thought… Gone the radical separation between him who loves and him who is beloved: God is there, and joy is boundless.’ Indeed, Bergson (1977, p. 209) even describes the union with God as a union with life itself: ‘the establishment of a contact… with the creative effort which life itself manifests. This effort is of God, if it is not God himself.’

23 It should however be noted that the language of ‘in’ in Bergson’s (1946, p. 132) account of how one ‘plunges’ or ‘immerses’ oneself ‘*in*’ real duration is not exactly found in the French original: ‘*Et plus nous nous y enfonçons, plus nous nous sentons approcher du principe dont nous participons*.’

24 Bergson’s opposition to the traditional theological conception of divine immutability is most powerfully formulated in his critique of the ‘motionless’ ‘God of Aristotle’ in *The Two Sources* (Bergson, 1977, pp. 230–233; cf. Bergson, 1946, pp. 34–35). Bergson’s emphasis on infinite mutability is very much related to the morality or ethos of ‘openness’ he envisions throughout *The Two Sources*. For a discussion of the significance of Bergson’s notion of ‘the open’, see Worms, 2012.

25 Cf. Deleuze, 1988, pp. 77–78: ‘If things are said to endure, it is less in themselves or absolutely than in relation to the Whole of the universe in which they participate insofar as their distinctions are artificial… there is, as it were, a mysterious participation of things in our duration… this participation in our duration would be explained by things belonging to the Whole of the universe… There is only a single time, a single duration, in which everything would participate, including our consciousnesses, including living beings, including the whole material world.’ The language of ‘participation in duration’ is also found in A. W. Moore’s (2022, p. 218) characterisation of Bergson’s metaphysics: ‘*Everything* participates in duration. Everything—including every material thing—participates in spirituality.’

26 Bergson’s (1972, p. 964) 20 February 1912 letter to Fr de Tonquédec.

27 Bergson’s (1972, p. 766) 12 May 1908 letter to Fr de Tonquédec.

28 For a contrary defence of Bergson from a Catholic commentator, see Chevalier, 1928, pp. 262–331.

29 Cf. Ansell-Pearson, 2018, pp. 19, 94, 162: ‘[Bergson’s] conception of duration, which is that of a “becoming” that flows out of previous forms while always adding something new to them, is very different from Spinoza’s conception of the “one complete Being” which manifests forms… Indeed, on one level it is possible to read *Creative Evolution* as an attempt to refute Spinoza and dispel the entrancing effect his logical conception of reality has over modern minds. For a system like Spinoza’s, Bergson notes, true or genuine being is endowed with a logical existence… Spinozism is an attempt to make vanish “the mystery of existence”… and instead of making actual observations of nature, the philosopher advances a logical system in which at the base of everything that exists is a self-positing being dwelling in eternity.’ With this refutation of Spinoza’s metaphysical postulation that all beings exist in a ‘static’ or even ‘lifeless’ God, Bergson’s attitude to Spinoza resembles Schelling’s in his famous *Freiheitsschrift*. I would like to thank one of the anonymous reviewers for this observation.

30 Bergson’s (1972, pp. 963–64) 20 February 1912 letter to Fr de Tonquédec.

31 Cf. Ansell-Pearson, 2018, p. 140: ‘Bergson’s spiritualism is unique since it conceives God as life: the divine force is the creative energy at work in the evolution of life.’

32 See Ansell-Pearson, 2019. On the correlation between the ethical and the metaphysical aspects of Spinoza’s *Ethics*, see Carlisle, 2021. Note also that the attainment of ‘joy’ is key to both Bergson’s and Spinoza’s conceptions of the task of philosophy. See Bergson, 1946, pp. 86, 106; cf. Carlisle, 2021, pp. 99–100.

33 Cf. Maritain, 1955, p. 58: ‘[Bergson’s philosophy is oriented] in the exact opposite direction from classical pantheism.’

34 This paper has focused primarily on Bergson’s work in the first two decades of the twentieth century. But it is worth noting that later in *The Two Sources*, Bergson commends ‘religion’ over ‘philosophy’ as a practice of thinking. See Ansell-Pearson, 2018, 123, 137: ‘one of the reasons why [Bergson] privileges religion over philosophy is because he sees it as a domain of action and creation… Bergson holds religion in its dynamic aspect to be superior to philosophy. This is because Bergson thinks philosophy, which is a species of intelligence, is bound up with contemplation and not action.’
